# Hydrogen Peroxide: Its Role in Plant Biology and Crosstalk with Signalling Networks

**DOI:** 10.3390/ijms19092812

**Published:** 2018-09-18

**Authors:** Martin Černý, Hana Habánová, Miroslav Berka, Markéta Luklová, Břetislav Brzobohatý

**Affiliations:** 1 Department of Molecular Biology and Radiobiology, Faculty of AgriSciences Mendel University in Brno, 613 00 Brno, Czech Republic; habanova.ha@gmail.com (H.H.); miroslavberka94@gmail.com (M.B.); luklovam@gmail.com (M.L.); brzoboha@ibp.cz (B.B.); 2 Phytophthora Research Centre, Faculty of AgriSciences, Mendel University in Brno, 613 00 Brno, Czech Republic; 3 CEITEC—Central European Institute of Technology, Faculty of AgriSciences Mendel University in Brno, 613 00 Brno, Czech Republic; 4 Brno Ph.D. Talent, South Moravian Centre for International Mobility, 602 00 Brno, Czech Republic; 5 Institute of Biophysics AS CR, 613 00 Brno, Czech Republic

**Keywords:** H_2_O_2_, plant hormone, signalling, growth and development, stress

## Abstract

Hydrogen peroxide (H_2_O_2_) is steadily gaining more attention in the field of molecular biology research. It is a major REDOX (reduction–oxidation reaction) metabolite and at high concentrations induces oxidative damage to biomolecules, which can culminate in cell death. However, at concentrations in the low nanomolar range, H_2_O_2_ acts as a signalling molecule and in many aspects, resembles phytohormones. Though its signalling network in plants is much less well characterized than are those of its counterparts in yeast or mammals, accumulating evidence indicates that the role of H_2_O_2_-mediated signalling in plant cells is possibly even more indispensable. In this review, we summarize hydrogen peroxide metabolism in plants, the sources and sinks of this compound and its transport via peroxiporins. We outline H_2_O_2_ perception, its direct and indirect effects and known targets in the transcriptional machinery. We focus on the role of H_2_O_2_ in plant growth and development and discuss the crosstalk between it and phytohormones. In addition to a literature review, we performed a meta-analysis of available transcriptomics data which provided further evidence for crosstalk between H_2_O_2_ and light, nutrient signalling, temperature stress, drought stress and hormonal pathways.

## 1. Introduction

Hydrogen peroxide, a chemical compound discovered by Louis Jacques Thenard a hundred years ago, has properties that could justify classifying it as a phytohormone. In nature, it can be of inorganic origin, for example, via reactions in the atmosphere [1] but H_2_O_2_ from this source has only an indirect effect on living organisms. Thenard was the first to discover not only that H_2_O_2_ decomposes into water but also that it can cause skin blistering at a high concentration. However, oxidative stress is not the sole effect of this molecule. It is an evolutionarily conserved signalling molecule and in plants, it has gained attention also for its role in the regulation of growth and development. Indeed, the number of H_2_O_2_-related research articles published each year has doubled since 2008, with Web of Science listing over 3000 plant science publications on this topic in the last five years. In this review, we summarize different aspects of H_2_O_2_-mediated responses in plants, starting with the sources, catabolism and transport of H_2_O_2_. We then describe mechanisms for its perception and discuss its role in plant signalling networks and its effects on plant growth and development.

## 2. Metabolism

**Figure 1:**
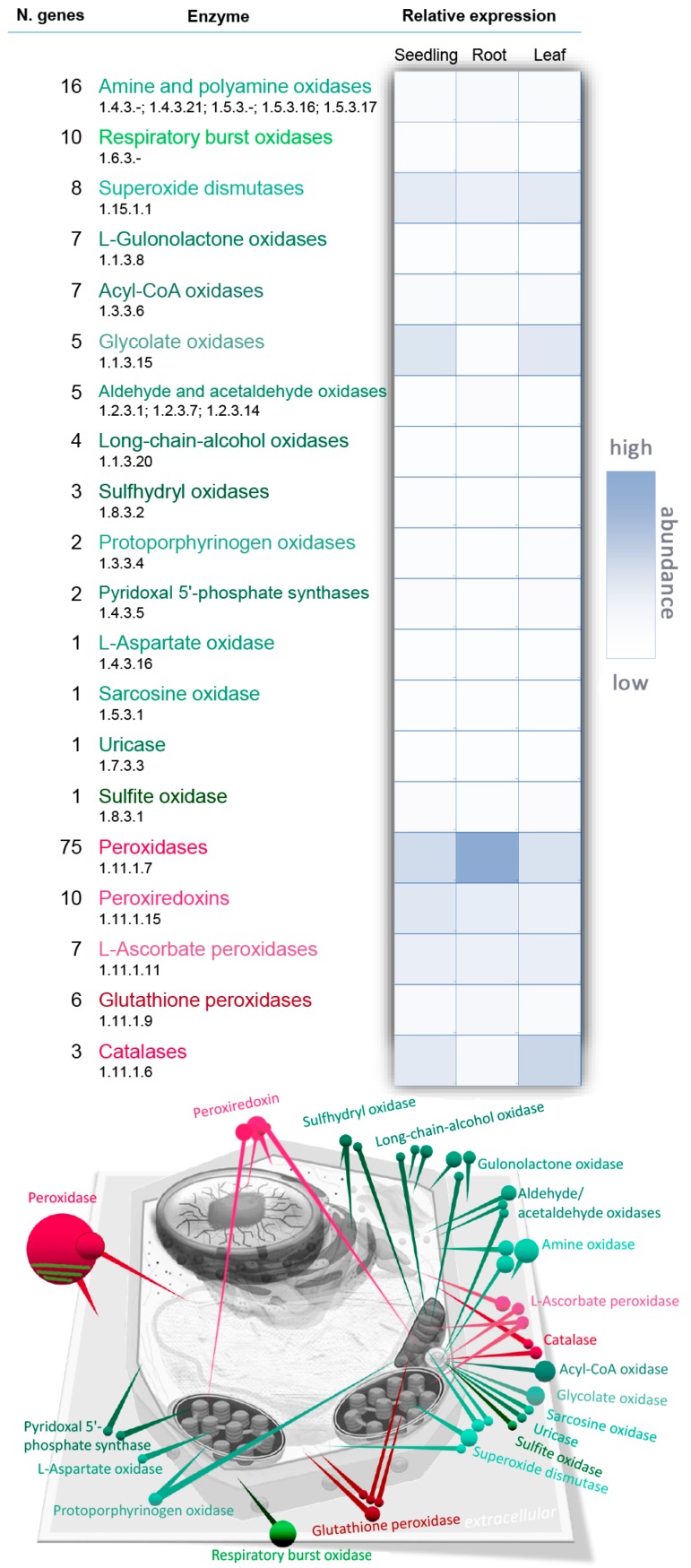
Key enzymes of hydrogen peroxide metabolism in plants. The list shows enzymes that directly catalyse hydrogen peroxide production or degradation in *Arabidopsis*, including the numbers of different isozymes, a comparison of relative gene expression profiles in seedlings, roots and shoots and the figure indicates subcellular localization. Colour coding: anabolic processes (green), catabolic processes (red), based on UniProt [16], SUBA 3.0 [17] and average gene expression profiles in 45, 24 and 7 NGS experiments for seedlings, leaf and root respectively (ThaleMine [18]).

Hydrogen peroxide H_2_O_2_ is a non-radical reactive oxygen species (ROS) and it, like singlet oxygen ^1^O_2_ and free radicals such as superoxide anion O_2_^−^ and hydroxyl radical ^•^OH, is one of the major members of the ROS family [2]. In contrast to other ROS, H_2_O_2_ is relatively stable, with a half-life of ms and its level in a plant leaf oscillates around 1 µmol per gram of fresh weight under natural conditions [3]. There are numerous routes, both enzymatic and non-enzymatic, for H_2_O_2_ production in plant cells. The key sources include photorespiration, electron transport chains and redox reactions in the apoplast [4,5]. The KEGG (Kyoto Encyclopedia of Genes and Genomes) database lists 150 classes of enzyme that produce or utilize hydrogen peroxide. Of these, only 29 enzymes encoded by 227 genes are annotated in *Arabidopsis* and the largest enzyme family formed by peroxidases has 75 entries (Figure 1, Supplementary tables). However, not all of these enzymes necessarily participate in peroxide metabolism in plants. For instance, a flavin-containing monooxygenase like YUC6 may produce hydrogen peroxide in the absence of its substrate but in vitro experiments indicate that in this case the uncoupled reaction represents less than 4% of the enzyme’s activity [6]. In contrast, mammalian flavin-containing monooxygenases are clearly a source of hydrogen peroxide [7]. The key enzymes that are involved in *Arabidopsis* H_2_O_2_ metabolism reside in the apoplast, peroxisome, chloroplast and mitochondria and they will be described in detail.

### 2.1. Electron. Transport Chains and Superoxide Dismutase

Under favourable conditions, the majority of intracellular H_2_O_2_ is produced from molecular oxygen by a stepwise reaction via a superoxide anion intermediate which undergoes enzymatic reduction to H_2_O_2_. Excessive energy and/or malfunctioning of chloroplast and mitochondrial energetic metabolism are key causes of superoxide anion generation in plant cells. In chloroplasts, superoxide anions are produced when the electron-transport chain of photosystem I is oversaturated by excessive irradiation and electrons are transmitted by the Mehler reaction to oxygen molecules [8]. The resulting superoxide anions are then converted to H_2_O_2_. This dismutation step is a pH-dependent non-enzymatic event (for details see for example, [9]) but cells also catalyse the process by means of superoxide dismutase (SOD) in order to rapidly remove the toxic superoxide radical. Besides photosystem I, H_2_O_2_ may also originate at the manganese-containing, oxygen-evolving complex which is the donor site of photosystem II and by the reduction of singlet oxygen or superoxide anions by photosynthetic electron transport chain components such as plastoquinol [10]. In seeds and non-photosynthetic parts of plants, the main sources of superoxide anion are coupled with the processes of cell respiration in mitochondria. Electron leakage occurs especially in complexes I, II and III and it is estimated that 1–5% of the oxygen entering the plant respiratory chain is converted into H_2_O_2_ [11,12,13]. The *Arabidopsis* genome encodes eight SOD isozymes which can be divided into three classes according to their metal cofactor (Fe^2+^, Mn^2+^, Cu^2+^). There are three chloroplastic Fe-SODs and two Mn-SODs localized in mitochondria. The Fe-SODs are considered to be the oldest in evolutionary terms but the two classes share structural similarities and can also be found in prokaryotes. In contrast, the Cu/Zn-SOD class, which has three isozymes in *Arabidopsis*, most likely emerged after oxygen saturated the atmosphere. It is specific to eukaryotes and can be present in different cell compartments [14,15].

### 2.2. NADPH Oxidase

The second largest group of H_2_O_2_-producing enzymes consists of the respiratory burst oxidases (Figure 1), which are also known as respiratory burst oxidase homologs (RBOHs) based on their homology to mammalian phagocyte NADPH oxidase (nicotinamide adenine dinucleotide phosphate oxidase). RBOHs, together with the type III cell wall peroxidases, are associated with the so-called “oxidative burst,” which is considered to be one of the main responses of plant cells to biotic or abiotic stress [19,20] but is also a crucial part of normal plant growth and development [21]. RBOHs are plasma membrane-localized proteins which oxidize cytosolic NADPH, transferring the released electron to O_2_ and producing superoxide which is then dismutated. In *Arabidopsis*, there are ten RBOH genes which are divided into three classes according to their tissue-specificity [22,23]. RBOHs are probably the best studied enzymatic ROS-generating system in plants and different regulatory mechanisms have been described. RBOHs undergo multiple post-translational modifications (PTMs), including S-nitrosylation and phosphorylation, that are required for enzyme activity and are regulated by calcium ions and phosphatidic acid [24,25].

### 2.3. Polyamine Oxidase

Hydrogen peroxide is an end product of oxidative degradation of amines and polyamine degradation is considered to be an especially important source of hydrogen peroxide in plants (e.g., [26]). Plant polyamines are catabolized by two distinct classes of amine oxidases, the flavin adenine dinucleotide (FAD)-dependent polyamine oxidases and the copper amine oxidases, of which there are, respectively, five and eight putative functional isozymes encoded by the *Arabidopsis* genome [27]. The copper amine oxidases oxidize primary amino groups, producing ammonia, H_2_O_2_ and an aminoaldehyde, whereas the polyamine oxidases oxidize the secondary amino groups and the reaction products depend on the catalytic mechanism and substrate specificity of a given isozyme. All five *Arabidopsis* polyamine oxidases are reportedly intracellular and oxidize the carbon on the exo-side of the N^4^ atom of spermine and spermidine to produce 1,3-diaminopropane, H_2_O_2_ and an aminoaldehyde [28]. Polyamines play an important role in plant tolerance of abiotic stress and at least part of this tolerance is associated with hydrogen peroxide production (see for example, review [29]). Furthermore, polyamines represent a direct link between H_2_O_2_ and hormonal pathways, as it has been shown that cytokinin increases the polyamine content of plants [30].

### 2.4. Peroxisomal Production of H_2_O_2_

Peroxisomal enzymes represent a major site of H_2_O_2_ production in a plant cell. In *Arabidopsis*, in addition to SOD and amine oxidases that are present in multiple compartments, peroxisomes contain acyl-CoA oxidases, glycolate oxidases, uricase, sulphite oxidase, aldehyde oxidase and sarcosine oxidase (Figure 1). Xanthine oxidase, which converts xanthine to urate and H_2_O_2_, can be also localized in peroxisomes [2] but a putative *Arabidopsis* homolog that preferentially accepts NAD^+^ as the electron acceptor [31] reportedly resides in the cytosol. A significant proportion of peroxisomal H_2_O_2_ originates during the beta-oxidation of long-chain fatty acids via acyl-CoA oxidase [32], which is an especially important process in germinating seeds that contain glyoxysomes, specialized peroxisome-like organelles. However, in photosynthetic tissues, the role of peroxisomes in H_2_O_2_ metabolism is predominantly via photorespiration reactions that may contribute up to 70% of the total production of H_2_O_2_ in a plant cell [33,34]. In this reaction, glycolate produced in chloroplasts is converted to glyoxylate by glycolate oxidase in peroxisomes. The *Arabidopsis* genome contains five genes encoding glycolate oxidase and their combined relative expression in photosynthetic tissues is the highest of all H_2_O_2_-producing enzymes (Figure 1). However, the actual levels of H_2_O_2_ in peroxisomes are kept in check by catalase and it is estimated that the peroxisomal H_2_O_2_ concentration is under 10 μM [35].

### 2.5. The H_2_O_2_ Scavenging System

Plant cells survive with H_2_O_2_ levels that would kill animal cells and the estimated endogenous H_2_O_2_ content of plant cells is usually much higher than that found in animals and bacteria [36]. H_2_O_2_ accumulation increases the probability of hydroxyl radical production via the Fenton reaction and this would cause significant oxidative damage to cellular structures if it were not for the presence of a highly efficient antioxidant system. Higher plants contain several types of peroxidases, including catalases, ascorbate peroxidases (APX), thiol-specific peroxidases and classical secretory plant peroxidase. Furthermore, non-enzymatic compounds like tocopherols, ascorbic acid and flavonoids and glutathione play significant roles in H_2_O_2_ scavenging [37,38]. The plastoquinone and ubiquinone pool also contribute to the ROS scavenging process as illustrated in recent reports [39,40]. In accordance, inhibition of enzymes that maintain the oxidized plastoquinone and ubiquinone pool, plastid terminal oxidases and mitochondrial alternative oxidases, respectively, stimulates H_2_O_2_ production [41,42].

### 2.6. Catalases

Though catalase belongs to the peroxidase family, it is usually considered separately due to its unique ability to convert two molecules of H_2_O_2_ into water and molecular oxygen without the need for any reductant. This heme-containing enzyme is first oxidized to a high-valence iron intermediate, which is then reduced by a further reaction with H_2_O_2_ [43]. Under specific circumstances, the intermediate may also react with a different substrate and catalase may oxidize donors such as alcohols or phenols. Catalase has a high turnover rate but a low substrate affinity, with a Km value in the millimolar range, a far greater concentration of H_2_O_2_ that that expected to be present in the cell [35]. As an illustration, the activity of a single molecule of rice catalase (k_cat_ 80,000; Km 100 mM) [44] would be equivalent to more than 2200% of tobacco APX (k_cat_ 1800; Km 0.022 mM) [45] at 100 mM H_2_O_2_ but to only 1% at concentrations below 1 µM H_2_O_2_, which would render catalase redundant. Of course, the constants determined in vitro may be misleading; the active form of catalase is a tetramer and it has been shown that, for example, PTMs may significantly affect the kinetics of a multimeric enzyme (e.g., [46]). Nevertheless, even though catalase activity has also been reported in the cytosol and mitochondria, its predominant localization is in peroxisomes, compartments with a high H_2_O_2_ concentration where its efficiency should be greatest (e.g., [47]). There are three functionally conserved classes of catalase with different spatial and developmental localizations in plants. For example, in tobacco catalase class I detoxifies H_2_O_2_ produced in photorespiration reactions, class II is localized in the vascular system and class III is present predominantly in flowers and fruits [48].

### 2.7. Ascorbate and Thiol-Specific Peroxidases

APX and glutathione peroxidases belong to the most important group of intracellular peroxidases [49]. Several types of APX have been described in plants; they include soluble enzymes in the cytosol, chloroplast and mitochondria and membrane-bound peroxidases in peroxisomes, glyoxysomes and thylakoids [50]. APX is the first enzyme in the so-called ascorbate-glutathione cycle, which includes monodehydroascorbate reductase, dehydroascorbate reductase and glutathione reductase and reduces H_2_O_2_ and regenerates ascorbate via NAD(P)H [49]. The *Arabidopsis* genome encodes seven different APX isozymes and as indicated above, APX may be more important than catalase for H_2_O_2_ metabolism. Indeed, it has been shown that in the absence of cytosolic APX1, the entire chloroplastic H_2_O_2_-scavenging system in *Arabidopsis* collapses, H_2_O_2_ levels increase and protein oxidation occurs [51]. The thiol-specific peroxidases peroxiredoxins and glutathione peroxidases detoxify a broad spectrum of peroxide substrates [8]. However, recent evidence from *S. cerevisiae* indicates that this could be a secondary role and that thiol peroxidases perceive and transfer oxidative signals to signalling proteins and regulate transcription [52]. In plants and bacteria, six groups of peroxiredoxins are recognized on the basis of differences in sequence, structure and positions of conserved cysteinyl residues [53].

### 2.8. Peroxidases (Class III)

Peroxidases are by far the most abundant family of enzymes in H_2_O_2_ metabolism (Figure 1). These so-called class III peroxidases probably have a correspondingly diverse range of functions, of which only a few, in certain plant species, have been revealed (see for example [54,55] for details). From the point of view of this review, it is important to note that the class III peroxidases participate not only in H_2_O_2_ catabolism via oxidation of phenolic compounds but also in producing it via an oxidative cycle using apoplastic reductants. For instance, it has been shown that in *Arabidopsis* cell culture they contribute to ca. 50% of the H_2_O_2_ produced during the oxidative burst in pathogen defence [56]. Class III peroxidases can be found in vacuoles but the majority are apoplastic or associated with cell walls in the apoplast as they play a key role in maintaining cell wall integrity by catalysing its cross-linking and loosening, lignification and suberization [57].

## 3. Transport

Normal levels of H_2_O_2_ leaf extracts are reported to be in the μmol per gram of fresh weight range but they may significantly vary within the same plant [3]. For instance, localization of hydrogen peroxide in different regions of the leaf reveals a pattern of increasing accumulation from the base to the leaf tip [58]. There is no clear evidence for long distance transport of H_2_O_2_ but it is the least reactive ROS and this allows it to travel at least among neighbouring cells or cellular compartments and to serve as an important signalling molecule [59]. Thus, if it is able to escape the H_2_O_2_-scavenging mechanisms described above and is not reduced to the highly reactive hydroxyl radical, it may freely diffuse from the site of its generation and reach its putative target. Questions of how it overcomes the competing H_2_O_2_-scavengers that prevent the targeted oxidation of redox-regulated proteins are still not fully answered [60] but it is now clear that transport mediated by simple diffusion would not explain, for example, rapid stress-induced transfer of H_2_O_2_ generated in apoplast by NADPH oxidases into cytosol and that a H_2_O_2_-specific transporter or channel must therefore exist.

### Peroxiporins

Henzer and Steudle found that treatment with HgCl_2_ (an aquaporin activity inhibitor) caused a rapid decrease in H_2_O_2_ and water influx and they postulated the existence of an aquaporin subclass, peroxiporins [61]. The similarity of H_2_O_2_ to the water molecule indicates that aquaporins could have such a function. Plant aquaporins are recognized as multifunctional proteins transporting not only water but also many other small uncharged molecules (e.g., CO_2_ and nutrients) and they thus play a role in the regulation of plant growth and development and in responses to a wide range of stresses. Aquaporins belong to the ancient superfamily of major intrinsic proteins (MIPs) and are present throughout living organisms with the exception of some Archea and bacteria [62]. Plant aquaporins are divided into five subfamilies: plasma membrane intrinsic proteins (PIPs), tonoplast intrinsic proteins (TIPs), nodulin26-like intrinsic proteins (NIPs), small basic intrinsic proteins (SIPs) and uncategorized intrinsic proteins (XIPs). The latter two groups, which were discovered more recently, are not present in some plant species [63]. H_2_O_2_ has a higher polarity than water and thus not all aquaporins are peroxiporins. For instance, Hooijmaijers et al. employed heterologous expression of all 13 *Arabidopsis* PIPs in yeast and found that only five of them inhibited yeast growth in the presence of H_2_O_2_ [64]. Since the first report of H_2_O_2_ transport by an aquaporin appeared, this phenomenon has been studied in diverse plant species, including maize [65], rice and barley [66], *Arabidopsis* [64,67,68,69], tulip [70], tobacco, potato and tomato [71]. Kim and Steudle (2009) suggested the occurrence of feedback regulation in aquaporin-facilitated H_2_O_2_ transport, based on the observed inhibition of aquaporin transport capacity after H_2_O_2_ treatment [72]. Further studies showed that this inhibition may occur indirectly by the internalization of aquaporin into vesicles that is caused by the change in the phosphorylation status of aquaporins [73,74]. Hooijmaijers et al. (2012) also found that H_2_O_2_ treatment can alter aquaporin expression, indicating a feedback loop between H_2_O_2_ concentration and peroxiporin expression [64].

## 4. Signalling

It has been widely reported that H_2_O_2_ effects are dose-specific and that at low concentrations it serves as a signalling molecule. Despite H_2_O_2_ being rapidly removed by protective enzymes, the scavenging mechanisms are less effective at concentrations of around 10 nM, enabling H_2_O_2_ to be a second messenger [59,75]. In general, proteins are primary targets of all oxidative species and there are two modes of action by which H_2_O_2_ is perceived: direct oxidation of amino acid residues or reaction with reactive intermediates (e.g., [76]). The latter represents an indirect effect mediated via peroxide decomposition products (hydroxyl radical and singlet oxygen) and is usually considered to be a non-specific oxidative stress response. However, it has been shown that the transcription factor PerR, a major regulator of the peroxide inducible stress response in bacteria, senses H_2_O_2_ via this pathway, employing metal-catalysed histidine oxidation [77]. The complexity of ROS-mediated processes in plants somewhat limits our understanding of H_2_O_2_ signalling circuits and the present state of this understanding lags far behind that for bacteria, yeasts or mammalian cells. For instance, the ratio of superoxide radical to hydrogen peroxide may regulate the respiratory chain in mitochondria [78] and it is believed that the ratio of singlet oxygen plus superoxide radical to hydrogen peroxide determines the activation of cell death programs [79]. Some mechanisms have been conserved during evolution, whereas others seem to be plant-specific. Here, we summarize the main circuits that have been found to operate in plants.

### 4.1. Oxidation of Cysteine Residues

Targets of direct oxidation are predominantly cysteinyl residues and reactive thiol side chains can act as sensors or switches in both signal transduction and regulation of enzyme activity [76]. Depending on H_2_O_2_ concentration a cysteinyl residue can react to undergo several reversible or irreversible modifications, starting with sulfenic acid, which is highly reactive and reacts with other proximal thiolates resulting in the formation of inter/intramolecular disulphide bonds or S-glutathionylation. The reduction of disulphide bonds and the removal of glutathione are regulated by members of the thioredoxin and glutaredoxin enzyme families. Sulfenic acid can be also further oxidized by H_2_O_2_ to sulfinic or even sulfonic acid [76,80]. Some signalling models predict that a hypothetical receptor may undergo successive oxidation steps and that each step would correspond to a physiological response but it remains to be seen whether such a receptor exists. Experiments carried out in vitro have shown that the rate of reaction of hydrogen peroxide with cysteine is relatively low but this does not apply to H_2_O_2_-scavenging enzymes. The reaction of the cysteinyl residue in peroxiredoxin has an apparent second order rate constant seven orders of magnitude higher than that for cysteinyl in BSA [81] and Marinho et al. calculated that the H_2_O_2_ concentration needed for a peroxiredoxin-mediated response time of 5 min is as low as 0.2 nM [82]. The thiol-specific peroxidases thus act as receptors and, upon oxidation, interact with and oxidize effector proteins, forming a redox relay. For example, *Arabidopsis* glutathione peroxidase functions as both a redox transducer and a scavenger in stomatal closure [83]. Key enzymes in photosynthesis and carbohydrate metabolism are oxidized in response to H_2_O_2_, including RuBisCO, phosphoribulokinase, glyceraldehyde-3-phosphate dehydrogenase, transketolase and sedoheptulose-1,7-bisphosphatase [84]. It is very likely that this is also a redox relay mediated by peroxiredoxins present in the chloroplast but evidence for this is lacking.

### 4.2. Oxidation of Methionine Residues

Methionine, the second proteinaceous sulphur-containing amino acid, is usually not considered to be a regulatory target in H_2_O_2_ signalling but its first oxidized form (methionine sulfoxide) is the product of a PTM that can be reversed via the action of a specific reductase [76]. The fact that this enzyme increases H_2_O_2_ tolerance indicates that methionine residues have a role at least in the H_2_O_2_-induced stress response [85]. Jacques et al. studied protein methionine sulfoxide dynamics in catalase knock-out *Arabidopsis* and found that 51 proteins were significantly more oxidized compared to wild-type. They also demonstrated that the activity of glutathione S-transferase is reduced upon methionine oxidation [86].

### 4.3. Other Protein PTMs

It should be noted that the direct effect of H_2_O_2_ on protein PTMs is not limited to cysteine or methionine residues. In fact, the presence of oxidative PTMs has been shown to interfere with other PTMs close to the oxidized site [87]. An alteration in the PTM pattern can play a crucial role in signalling. The well-known regulator TP53, which participates in mammalian H_2_O_2_ signalling, has to integrate a complex network of PTMs [82]. Its *Arabidopsis* orthologue SOG1 (suppressor of gamma response 1) is hyperphosphorylated in response to ROS and it has been proposed that H_2_O_2_ regulates its hyperphosphorylation, ultimately leading to cell cycle regulation [88]. Examples from mammalian systems also indicate that PTM by ubiquitination and targeted protein degradation is key to the H_2_O_2_ response [82]. However, our knowledge about its role in plant H_2_O_2_ circuits is limited. It has been found that UPL5 ubiquitin ligase mediates degradation of the transcription factor WRKY53 [89] but there are more than 1500 E3 enzymes in *Arabidopsis* and this, together with extensive crosstalk with phytohormonal networks (which all to some extent converge on the proteasome) [90], represents a substantial obstacle to the elucidation of H_2_O_2_ signal transduction.

### 4.4. Transcription Factors

#### 4.4.1. HsfA

Heat-shock transcription factors are transcriptional activators that, once trimerized, specifically bind *cis*-elements called heat shock elements, palindromic DNA sequences that are found in the promoters of heat stress-inducible genes of all eukaryotes, including that encoding APX, the major catabolic enzyme in *Arabidopsis* H_2_O_2_ metabolism [91,92]. The trimerization mechanism requires intramolecular disulphide bonds and it can be directly induced by H_2_O_2_ (reviewed in, for example, [93]). In *Arabidopsis*, HsfA2 was found to be involved in H_2_O_2_ signalling and it was shown that both its transcript and the transcript levels of its target genes were induced by treating with exogenous H_2_O_2_ [94].

#### 4.4.2. NAC Domain-Containing Protein

NAC (No apical meristem *Arabidopsis* transcription activation factor Cup-shaped cotyledon) domain-containing proteins constitute one of the largest transcription factor families in plants and they are involved in multiple developmental and physiological processes, including senescence and abiotic stress responses. Multiple genes of this family have been found to be upregulated in response to H_2_O_2_ [95] treatment and it has been suggested that NAC042 (JUB1) functions as a modulator of cellular H_2_O_2_ levels [96]. *NAC059*-dependent gene expression was triggered after H_2_O_2_ treatment [97] indicating that NAC could be a primary target of H_2_O_2_. Furthermore, two transcription factors (NAC013 and NAC017) that apparently shuttle between the nucleus and endoplasmic reticulum membrane mediate redox-related retrograde signalling [98,99].

#### 4.4.3. Mediators of RNA Polymerase

In yeast and mammals, an RNA polymerase inhibitor localized in cytosol is activated by H_2_O_2_ through the thioredoxin system and translocated into the nucleus [82]. Its putative orthologue in *Arabidopsis* is not known to be a H_2_O_2_-responsive protein but mediators of RNA polymerase II have been found to be upregulated in response to H_2_O_2_, including MED37C [94]. Shaikhali et al. showed that members of this family readily form oligomers in vitro via intramolecular disulphide bonds [100] and showed that root growth in the knock-out mutant *med32* was significantly less affected by H_2_O_2_ than that in wild-type plants [101].

#### 4.4.4. WRKY and ZAT (Zinc finger of *Arabidopsis thaliana*) Transcription Factors

There are 74 WRKY amino acid signature sequence-containing transcription factors in *Arabidopsis* that contain four-stranded β-sheet WRKY DNA binding domain/s ca 60 amino acids in length and zinc-finger motifs [102]. Like Nascent polypeptide-Associated Complex NAC domain-containing proteins, these transcription factors participate in stress-related responses and some have been found to be upregulated in response to H_2_O_2_. WRKY30 and WRKY53 were found to be upregulated in response to ozone and H_2_O_2_ exposure, with WRKY53 being much more responsive to H_2_O_2_ than WRKY30 and vice versa for ozone [103]. WRKY46 was upregulated by H_2_O_2_ [104] and WRKY70 is a putative interactor of the H_2_O_2_-responsive zinc finger protein ZAT7 [105]. ZAT12, another H_2_O_2_-responsive transcription factor, was proposed to mediate iron uptake control via its interaction with the FIT protein and with H_2_O_2_ as a signal in iron deficiency responses [106]. The present evidence indicates that WRKY transcription factors and ZAT zinc finger proteins participate in responses to H_2_O_2_ but a more detailed analysis of WRKY/ZAT-mediated transcription is needed in order to test the hypothesis that they play a role as the primary target. The fact that ZAT12 and ZAT5 respond positively to both ascorbate and H_2_O_2_ [107] indicates that this is probably not the case, at least for the ZATs.

### 4.5. Calcium Ions

Calcium ions play a key role in a vast array of signalling pathways in plants (e.g., [108]). Ca^2+^ is a second messenger like H_2_O_2_ and multiple characterized cascades require their combined effect, for example, via the opening of H_2_O_2_-dependent Ca^2+^ channels [109,110,111]. The Ca^2+^-binding protein calmodulin is an activator of catalase [112] and calmodulin-binding transcription activators have been found to be upregulated by H_2_O_2_. BT2, another calmodulin-binding protein which is upregulated by H_2_O_2_, is also part of an E3 ligase complex [113,114]. Moreover, Ca^2+^-dependent phosphorylation activates NADPH oxidases (e.g., [115]) and plays a role in the so-called ROS-Ca^2+^ hubs described in Section 5.11.

## 5. H_2_O_2_ in Growth and Development

**Figure 2:**
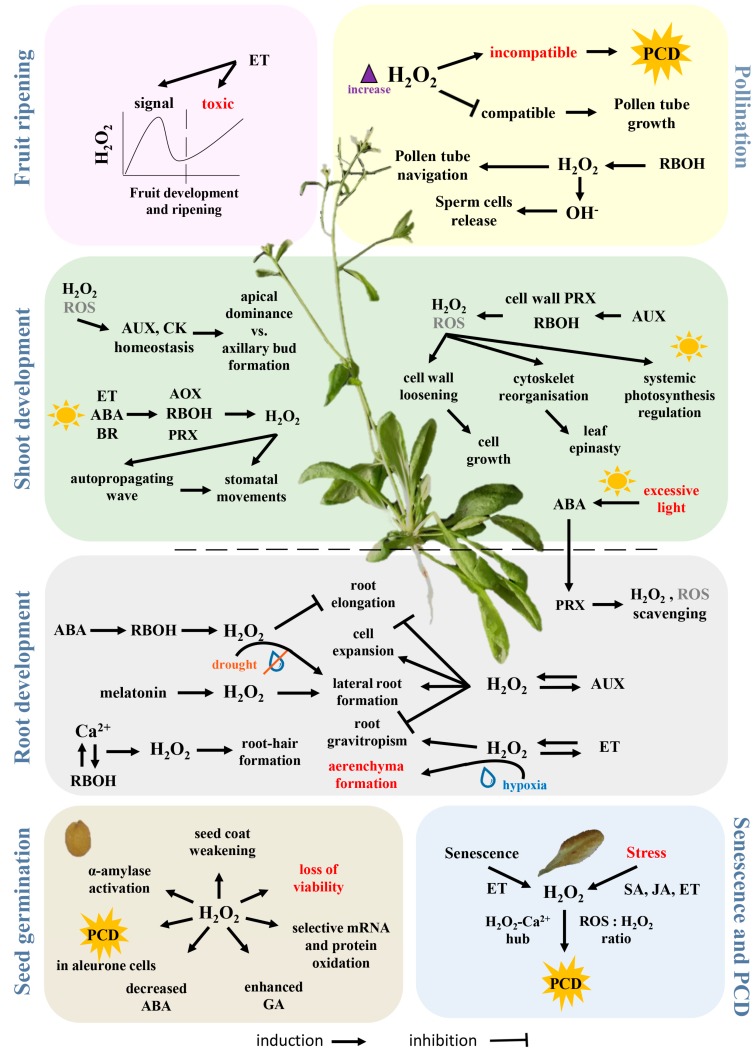
Hydrogen peroxide-mediated processes in plant growth and development. This figure summarizes our present-day knowledge about the role of H_2_O_2_ in the life of plants as described, with references, in Section 5. ABA—abscisic acid, AUX—auxin, BR—brassinosteroids, ET—ethylene, GA—gibberellins, SA—salicylic acid, JA—jasmonic acid, AOX—amine oxidases, PRX—peroxidases, RBOH—NADPH oxidases, PCD—programmed cell death. The water droplet shape indicates flooding and absence of water, for hypoxia and drought, respectively.

The role of H_2_O_2_ in the life of plants is illustrated in Figure 2 and outlined in the following text, which presents examples from different developmental stages.

### 5.1. The Crosstalk between H_2_O_2_ and Phytohormones

The first genome-wide analyses of plant H_2_O_2_ signalling revealed a connection between ethylene and H_2_O_2_. Ethylene signalling is induced in response to H_2_O_2_ accumulation [116] but the ethylene receptor ETR1 itself perceives H_2_O_2_ directly in an ethylene-independent manner that does not require its kinase domain [117]. ROS is a key component of phytohormonal signalling networks and does not only mediate stress-related pathways. From the proteome-wide point of view, catalases, peroxiredoxins, disulphide isomerases and thioredoxins have been detected at high frequencies in phytohormone-responsive proteomics studies and APX, glutathione S-transferase and class III peroxidase were found at least once in all reported hormone-responsive proteomes; see Table 1 [90]. H_2_O_2_ mediates hormonal homeostasis (e.g., auxin conjugation [118] and degradation [119]) but enzymes involved in hormone metabolism may produce H_2_O_2_. These include abscisic acid aldehyde oxidases, enzymes that catalyse the final step in abscisic acid biosynthesis producing H_2_O_2_ in the process. Auxin aldehyde oxidases are also present in *Arabidopsis* but it is not clear to what extent these enzymes contribute to auxin metabolism [120]. Furthermore, monooxygenases may catalyse a H_2_O_2_-producing side reaction, as illustrated above for the auxin biosynthetic enzyme YUC6, which is encoded by a member of a multigene family in *Arabidopsis*. Similar enzymes are present in the pathways of cytokinin metabolism (hydroxylases cytochrome P450 735A1 and 735A2), gibberellin and brassinosteroid biosynthesis (ent-kaurene oxidase, ent-kaurenoic acid oxidase) and abscisic acid metabolism (hydroxylases cytochrome P450 707A1-707A4). Our comparison of expression profiles revealed that multiple hormonal metabolism genes share patterns of expression with those of H_2_O_2_ metabolism; the former include *ABA4* (31 similar patterns), tryptophan aminotransferases *TAR3* (29) and *TAR4* (29), methyl esterase *MES1* (28), cytokinin biosynthetic genes *IPT2* (26), *IPT6* (22) and *LOG2* (24), ethylene metabolism genes *ACO2* (23) and *ACS4* (20) and Ent-copalyl diphosphate synthase *GA1* (27) (see Table 2 and Supplementary Materials for details).

### 5.2. Light Signalling

Light signal transduction is involved in H_2_O_2_ metabolism and/or signalling. It has been demonstrated that blue-light perception by cryptochrome is directly coupled with H_2_O_2_ generation [140,141,142]. It has also been proposed that phytochrome B modulates homeostasis of reactive oxygen species in roots via synthesis and transport of abscisic acid [143]. Our comparison of expression profiles revealed that genes participating in light signalling share patterns of expression with H_2_O_2_ metabolism genes; the former include *MED25* which acts in the repression of phytochrome B-mediated light signalling (26 similarities), *COP1* (32), *phytochrome A* (29), *PIF1* (28), *phytochrome* B (26), *phytochrome C* (25) and *cryptochrome 1* (18) (see Table 2 and Supplementary Materials for details).

### 5.3. Dry Seed

The majority of plants from temperate climate zones produce so-called orthodox seeds which pass through a phase of intensive desiccation and in this state, they are able to survive for periods ranging from months to tens of years (or even hundreds of years in some cases) [144]. The quiescent state limits enzymatic activity to a minimal level but H_2_O_2_ and other ROS are still produced and can be accumulated during seed ageing. H_2_O_2_ accumulation in seeds may cause significant damage to storage molecules and loss of viability but the degree to which it accumulates and the sensitivity to oxidative damage is species specific. For example, H_2_O_2_ does not accumulate in *Brassica napus* seeds [145].

### 5.4. Germination

Seed germination is defined as a three-phase process, starting with rapid water intake and ending with seed coat rupture, usually by radicle protuberance. In imbibed and germinating seeds, high levels of H_2_O_2_ are produced mainly as a product of intensive metabolism in mitochondria, peroxisomes and glyoxysomes but also by NADPH oxidases and through lipid peroxidation [146]. Though seeds contain both enzymatic and nonenzymatic ROS scavenging machinery to prevent oxidative damage [147], H_2_O_2_ is also needed to remove mechanical and hormonal barriers that inhibit germination. H_2_O_2_ promotes endosperm weakening [148,149] and triggers an increase in gibberellin biosynthesis and a decrease in abscisic acid levels [150,151,152,153,154]. It also mediates selective oxidation of mRNA and proteins [155,156], for example irreversible carbonylation of storage proteins that enables their rapid mobilization via proteasomes [157]. Another key aspect of seed germination, especially in cereals, is the activation of α-amylase and the promotion of programmed cell death (PCD) in the aleurone layer. Here, H_2_O_2_ is produced by NADPH oxidase and it functions via interplay with DELLA proteins (proteins with the highly conserved amino acid sequence motif DELLA), key components of the gibberellin signalling pathway [158,159,160]. In many respects, the role of H_2_O_2_ in germination is similar to that of a growth regulator and studies of exogenous H_2_O_2_ application have demonstrated that its effect is dose dependent [161,162,163].

### 5.5. Root Development

Ample evidence showed that ROS regulates root development via NADPH oxidases [111,164,165]. The phytohormone that is key to the regulation of root growth is auxin, which is well known to mediate changes in H_2_O_2_ levels and thus promote cell growth and lateral root formation [166,167,168]. However, a recent study indicated that in mediating the induction of lateral roots, H_2_O_2_ acts downstream of melatonin, an auxin-like indoleamine compound [169]. Root tip growth is also known to be affected by H_2_O_2_ [170,171]. Polar auxin transport seems to regulate H_2_O_2_-induced root gravitropism [163] and exogenous H_2_O_2_ treatment can disrupt this sensing, probably due to a change in auxin receptor distribution [172]. Abscisic acid inhibits primary root growth by activating NADPH oxidases and thus reducing auxin sensitivity [173] and a RBOH was proposed to interact with abscisic acid in the regulation of lateral root growth in *Arabidopsis* under drought stress [174]. H_2_O_2_-mediated root growth in response to stress was also found in cucumber [175], cotton [176] and rice [177].

### 5.6. Shoot Development

Shoot growth and development of shoot architecture are driven by phytohormones, especially auxin and cytokinin, levels of which are highly correlated with environmental conditions. Auxin participates in cell growth by inducing cell wall peroxidases (peroxidases class III) and NADPH oxidases to produce ROS and promote cell wall loosening and further cell elongation [178,179]. H_2_O_2_ has been reported to mediate apical dominance [180], photosynthesis [181] and leaf epinasty [182].

### 5.7. Stomatal Movement

Stomata are formed as gaps between pairs of guard cells and changes in guard cell turgor mediate the opening and closure of the stomatal pore. Stomatal closure is an example of rapid leaf-to-leaf communication mediated by ROS (e.g., [183]). Under conditions of excessive irradiation, an autopropagating wave of ROS rapidly transfers a signal to leaves that are not directly exposed to light and initiates stomatal closure. This closure can be induced by multiple stimuli, including brassinosteroids [184,185], strigolactones [186], jasmonic acid and salicylic acid [187], CO_2_ [188], ethylene [189,190], glucose [191] and interactions among them [192]. The best-described mechanism is that mediated by abscisic acid that recruits calcium ions, nitric oxide (NO), H_2_O_2_ and regulatory phosphorylation [193,194]. Guard cells generate H_2_O_2_ by means of amine oxidases [195], peroxidases and RBOHs [196,197]. The activity of RBOHs is regulated by Ca^2+^ binding [198] and phosphorylation by protein kinase OST1 (OPEN STOMATA 1) [199], which in turn is regulated by abscisic acid and interacts with a peroxiporin [200,201]. The overall H_2_O_2_-mediated machinery is much more complex. For example, hydrogen sulphide promotes H_2_O_2_ production by stimulating RBOH activity [202] but the presence of flavonols in guard cells and H_2_O_2_ scavenging inhibits stomatal closure [203] and it has been shown that this flavanol accumulation is induced by 5-aminolevulinic acid [204,205].

### 5.8. Pollination

In generatively propagating plants, H_2_O_2_ and other ROS play a key role in pollen navigation and gametophyte fusion. Angiosperms have developed different reproductive barriers to avoid self-fertilization, one of the most widespread being self-incompatibility [206]. H_2_O_2_ level is elevated during the incompatible reaction, triggering PCD. In contrast, the compatible reaction decreases the level of H_2_O_2_ in the stigma and the development of the pollen tube is promoted. The further growth of and the penetration of the ovule by, the pollen tube is guided by quite complicated signalling machinery, including the FERONIA protein which modulates NADPH oxidase activity [207,208]. ROS accumulation, especially that of the hydroxyl radical which is largely generated from H_2_O_2_, is then crucial for pollen tube rupture and the release of sperm cells [209].

### 5.9. Fruit Ripening

The involvement of H_2_O_2_ in the ripening process is known but not fully understood. Huan et al. proposed that H_2_O_2_ acts as a signalling molecule in the middle stage of peach fruit development but that it serves as an important toxic molecule, stimulating lipid peroxidation and oxidative stress, during the late stage of fruit ripening [210]. Kumar et al. analysed ripening in tomato and found changes in the redox state during different stages of ripening with a significant increase of H_2_O_2_ at the so-called breaker point (defined by the initial change in fruit colour) [211]. The increase in H_2_O_2_ is most likely regulated by ethylene, the key regulator of fruit ripening that enhances respiration rate and ROS production [212].

### 5.10. Senescence and Cell Death

Senescence ultimately leads to the death of plant organs or whole plants. It is a multistep process by which the plant recovers and recycles valuable nutrient components that would otherwise be lost [213]. The role of H_2_O_2_ in plant senescence was investigated by Bieker at al., who showed time-dependent levels of H_2_O_2_ and H_2_O_2_-scavenging enzymes in senescent leaves [214]. In such tissues H_2_O_2_ mediates PCD together with stress phytohormones like ethylene [215] or salicylic acid [216]. H_2_O_2_ levels are transiently elevated at the initial point of leaf senescence and peak again during the terminal stage [217,218] and this accumulation is reportedly more pronounced inside interveinal tissue [219]. Furthermore, transgenic lines with lower H_2_O_2_ levels display delayed senescence [96,214].

### 5.11. Stress

The key phytohormones orchestrating plant stress responses are abscisic acid, salicylic acid, jasmonates and ethylene and all of these phytohormones employ H_2_O_2_ in their signalling cascades in an either upstream or downstream manner [220]. Putative markers of nutrient status, temperature stress and drought stress share patterns of expression with those of H_2_O_2_ metabolism (Table 2) and H_2_O_2_ has been implicated in cold acclimation [221], salt stress responses and salt stress tolerance [222,223,224] and hypoxia stress [225]. Important targets in these responses are RBOHs [177,226,227]. Recently, maintenance of acquired thermotolerance was found to be interlinked with generation of H_2_O_2_ by RBOHs [228] and these NADH oxidases also participate in H_2_O_2_ production in biotic interactions. Under pathogen attack, ROS accumulation is involved in PCD of infected and surrounding cells [229]. This hypersensitive response is orchestrated by the phytohormones ethylene, JA and SA (e.g., [214]) but high cytokinin levels also induce H_2_O_2_ accumulation [230]. H_2_O_2_ has been implicated in the susceptibility of *Brassica napus* to *Leptosphaeria maculans* [231], resistance to root-knot nematode in tomato [232], systemic virus resistance in *Nicotiana benthamiana* [233] and reduction of rot in postharvest citrus fruits [234]. In accordance, plants primed with H_2_O_2_ or with a higher basal level of H_2_O_2_ formation display enhanced resistance to stressors [42,235].

It is well established that a significant proportion of H_2_O_2_-mediated stress response originates from its decomposition products. This decomposition is enhanced by the presence of transient metal catalysts through the so-called Haber-Weiss reaction. It is widely postulated that this reaction accounts for the in vivo generation of the highly reactive hydroxyl radical, which is a prime cause of oxidative damage to biomolecules (e.g., [9,236]). The hydroxyl radical is one of the strongest oxidants known and reacts at nearly diffusion-limited rates near the site of its formation [237]. Besides its ability to damage anything in its close vicinity and generate further radicals, the hydroxyl radical seems to be a potent effector in calcium and potassium homeostasis, regulation of cell elongation and stress-induced cell death [111,238,239,240,241]. Furthermore, hydroxyl radical-mediated activation of calcium channels is also proposed to be a part of the so-called ROS-Ca^2+^ hub, the mechanism that is utilized to perceive and amplify signal. This self-amplifying system employs Ca^2+^-dependent phosphorylation of NADPH oxidases and promotes hydroxyl radical production that, in turn, stimulates Ca^2+^-influx and NADPH oxidases’ activity (see for example [242]). The ROS-Ca^2+^ hub is believed to be central to hypersensitive response, phytohormonal signalling or abiotic stress responses [115,158,243,244]

Organelles like chloroplasts or mitochondria are key cellular sensors of environmental fluctuations and integral parts of plant stress responses. They communicate information by signalling to nuclei via stress-triggered retrograde signals, including ROS (reviewed in Reference [245]). Recent reports show not only that H_2_O_2_ participates indirectly via ROS triggered signals but also that it can transfer from chloroplasts to nuclei and facilitate photosynthetic control over gene expression [246].

## 6. Conclusions

H_2_O_2_ represents a key signalling molecule, connecting the signalling pathways of multiple phytohormones and acting as a second messenger in response to diverse conditions modulating plant growth and development. Its dose-dependent effect on growth clearly indicates that H_2_O_2_ is a growth regulator but can we also refer to H_2_O_2_ as a putative phytohormone? It is produced and degraded by the plant in response to stimuli and it is perceived by specialized proteins and elicits a response at low nanomolar concentrations. However, the limiting factor is its transport. Though it can be readily transported within a single cell and exported to extracellular space, it is not believed to serve as a long-distance signal due to its low stability and the presence of H_2_O_2_ scavengers. Exogenous treatment with H_2_O_2_ elicits a response and H_2_O_2_ gradients are established in plant organs but it is believed that signal propagation is sequential and that H_2_O_2_ reaches only neighbouring cells [248]. In conclusion, the recent literature offers multiple examples that reveal H_2_O_2_ as a versatile mediator of molecular communication in plants and whether we classify it as a phytohormone or not, this does not change its importance in the life of plants. There are new perspectives emerging in the field of H_2_O_2_ research with tools being developed for the detection of low micromolar and even picomolar H_2_O_2_ concentrations [249,250] and it is likely that their eventual application in plant sciences will provide answers to some of our questions about H_2_O_2_ transport and concentration dynamics. Similarly, we may expect that increasing sensitivity in proteomics approaches combined with imaging or laser microdissection techniques (e.g., [251]) will reveal more H_2_O_2_ targets and their spatio-temporal distribution.

## Figures and Tables

**Table 1:** Proteins of hydrogen peroxide metabolism in *Arabidopsis* identified in phytohormone-responsive proteomics analyses. Based on a previously published overview of hormone-responsive proteins [90].

AGI	Protein Name (UniProt)	Relative Protein Abundance						
		Auxin	Abscisic Acid	Brassinosteroid	Cytokinin	Salicylic Acid	Jasmonate/Oxylipins	Strigolactone
AT1G05260	Peroxidase 3		down [121]					
AT1G06290	Acyl-coenzyme A oxidase 3						up [122]	
AT1G07890	L-Ascorbate peroxidase 1		down [121]		down [30,123,124]		up [125,126]	
AT1G08830	Superoxide dismutase [Cu-Zn] 1					up [127]		
AT1G20620	Catalase-3		up [128]		down [129]	up [130]	up [130]	
AT1G20630	Catalase-1		up [131]					
AT1G31710	Amine oxidase		down [121]					
AT1G44446	Chlorophyllide a oxygenase			down [132]				
AT1G65980	Peroxiredoxin-2B		down [121]				up [126]	
AT1G71695	Peroxidase 12		down [121]	down [132]				
AT1G77490	L-Ascorbate peroxidase T	up [133]						
AT2G18150	Peroxidase 15					up [127]		
AT2G22420	Peroxidase 17					up [127]		
AT2G26230	Uricase		down [121]					
AT2G28190	Superoxide dismutase [Cu-Zn] 2						up [134]	
AT2G30490	Trans-cinnamate 4-monooxygenase		up [131]					
AT2G43350	Probable glutathione peroxidase 3		down [121]					
AT3G06050	Peroxiredoxin-2F						up [134]	
AT3G10920	Superoxide dismutase [Mn] 1				down [135]	up [136]	down [126]	
AT3G11630	2-Cys peroxiredoxin BAS1				up [30,129]		up [125]	
AT3G14415	(S)-2-hydroxy-acid oxidase		down [125]			up [130]	up [125,130]	
AT3G14420	(S)-2-hydroxy-acid oxidase GLO1				up [30]	up [130]	up [126,130]	
AT3G26060	Peroxiredoxin Q, chloroplastic						up [134]	
AT3G32980	Peroxidase 32				down [30]	up [127]		
AT3G49120	Peroxidase 34		up [128,131]		down [30]	up [127]		
AT3G56350	Superoxide dismutase [Mn] 2					up [137]		
AT4G08390	L-Ascorbate peroxidase S		up [126]		down [30]			
AT4G08770	Peroxidase 37					up [127]		
AT4G08780	Peroxidase 38					up [127]		
AT4G15760	Monooxygenase 1							up [137]
AT4G16760	Acyl-coenzyme A oxidase 1	up [133]					up [122]	
AT4G25100	Superoxide dismutase [Fe] 1					up [127]	up [125]	
AT4G35000	L-Ascorbate peroxidase 3				down [30]			
AT4G35090	Catalase-2		up [131]		up/down [30,123]	up [130]	up [130]	
AT4G36430	Peroxidase 49					up [127]		
AT5G06290	2-Cys peroxiredoxin BAS1-like						up [126]	
AT5G14220	Protoporphyrinogen oxidase 2			up [132]	up [132]			
AT5G17820	Peroxidase 57		up [128]					
AT5G18100	Superoxide dismutase [Cu-Zn] 3					up [127]		
AT5G23310	Superoxide dismutase [Fe] 3			down [138]			down [122]	
AT5G49970	PYRIDOXINE/PYRIDOXAMINE 5′-PHOSPHATE OXIDASE 1						up [122]	
AT5G51100	Superoxide dismutase [Fe] 2		up [139]					
AT5G64120	Peroxidase 71		down [131]				up [122]	down [137]
AT5G65110	Acyl-coenzyme A oxidase 2			down [132]				

**Table 2:** Hydrogen peroxide metabolism genes have expression patterns similar to those of genes related to light signalling, nutrient status, temperature stress, drought stress and hormonal metabolism. Based on average gene expression profiles in stress-related experiments (ThaleMine [11]) and reference stress-related genes [247]. Numbers indicate the number of analysed genes (numbers in brackets) and the number of detected co-expressed genes (hydrogen peroxide metabolism/candidate signalling and metabolism genes). See Supplementary Materials for the full list of co-expressed genes.

	Nutrient Stress (142)	Temperature Stress (43)	Drought Stress (13)	Light Signalling (27)	Abscisic Acid Metabolism (16)	Auxin Metabolism (31)	Brassinosteroid Metabolism (13)	Cytokinin Metabolism (37)	Ethylene Metabolism (12)	Gibberellin Metabolism (23)	Jasmonate Metabolism (17)	Salicylic Acid Metabolism (9)	Strigolactone Metabolism (3)
Amine/polyamine oxidase (15)	11/40	4/6	1/1	7/16	6/5	5/6	5/5	10/15	5/4	4/7	3/5	2/1	0/0
Respiratory burst oxidase (10)	10/58	6/6	4/1	9/16	5/3	7/14	8/8	9/21	4/4	8/6	3/2	4/4	4/3
Superoxide dismutase (8)	7/46	5/5	0/0	4/13	5/2	5/9	4/6	5/15	2/3	5/10	1/3	2/2	0/0
L-Gulonolactone oxidase (7)	7/31	2/2	2/1	4/5	4/5	4/7	2/1	7/10	3/3	3/5	3/4	3/2	2/2
Acyl-coenzyme A oxidase (7)	5/19	4/6	4/2	4/14	4/3	6/9	4/2	5/11	0/0	5/7	6/6	1/1	2/2
Glycolate oxidase (5)	5/10	2/1	0/0	5/8	0/0	3/3	4/2	3/4	0/0	2/1	3/2	0/0	3/1
Aldehyde/acetaldehyde oxidase (5)	4/36	2/1	2/1	4/9	3/3	5/8	4/4	4/13	1/1	3/7	1/2	3/3	1/2
Long-chain-alcohol oxidase (4)	3/18	3/4	0/0	2/7	2/2	3/5	1/1	3/6	1/1	1/1	1/2	2/2	0/0
Sulfhydryl oxidase (3)	3/25	3/5	0/0	3/13	3/2	3/8	3/3	3/5	2/2	3/6	3/3	2/1	1/1
Protoporphyrinogen oxidase (2)	2/21	2/1	0/0	2/5	2/2	2/6	2/3	2/6	2/1	2/3	0/0	1/2	0/0
Pyridoxal 5′-phosphate synthase (2)	2/28	2/3	0/0	2/9	2/2	2/7	2/3	2/8	2/2	2/5	1/1	2/2	1/1
L-Aspartate oxidase (1)	1/5	1/1	1/1	1/1	0/0	0/0	0/0	0/0	0/0	1/1	0/0	0/0	1/1
Sarcosine oxidase (1)	1/1	0/0	0/0	1/1	0/0	1/1	0/0	0/0	0/0	0/0	0/0	0/0	0/0
Uricase (1)	1/6	1/1	1/1	1/3	1/1	1/1	1/1	1/1	0/0	0/0	1/1	0/0	1/1
Sulphite oxidase (1)	1/12	1/3	0/0	1/9	1/1	1/5	1/1	1/4	1/1	1/3	1/1	1/1	0/0
Peroxidase (73)	53/106	21/9	9/2	33/25	21/7	35/21	29/13	43/30	21/6	27/15	17/8	18/7	10/3
Peroxiredoxin (10)	8/43	4/7	1/1	5/14	5/3	3/7	3/5	6/15	4/4	7/12	2/4	4/3	0/0
L-Ascorbate peroxidase (7)	6/44	6/7	2/2	6/16	3/2	4/10	5/7	6/16	3/4	6/9	4/3	3/2	2/2
Glutathione peroxidase (6)	5/26	3/6	0/0	3/10	1/1	5/8	3/3	3/6	3/2	3/3	1/4	1/1	2/2
Catalase (3)	3/13	2/2	1/1	2/13	1/1	3/5	2/3	3/8	0/0	3/3	2/4	0/0	2/1

## Supplementary Materials

Supplementary materials can be found at http://www.mdpi.com/1422-0067/19/9/2812/s1.
