# Supplementary figures and images for: Hydrogen Peroxide: Its Role in Plant Biology and Crosstalk with Signalling Networks
Source: Int J Mol Sci. 2018 Sep 18;19(9):2812. doi: 10.3390/ijms19092812 (PMC6163176; doi:10.3390/ijms19092812)

Figure S1. Supplementary figure to Table 2.

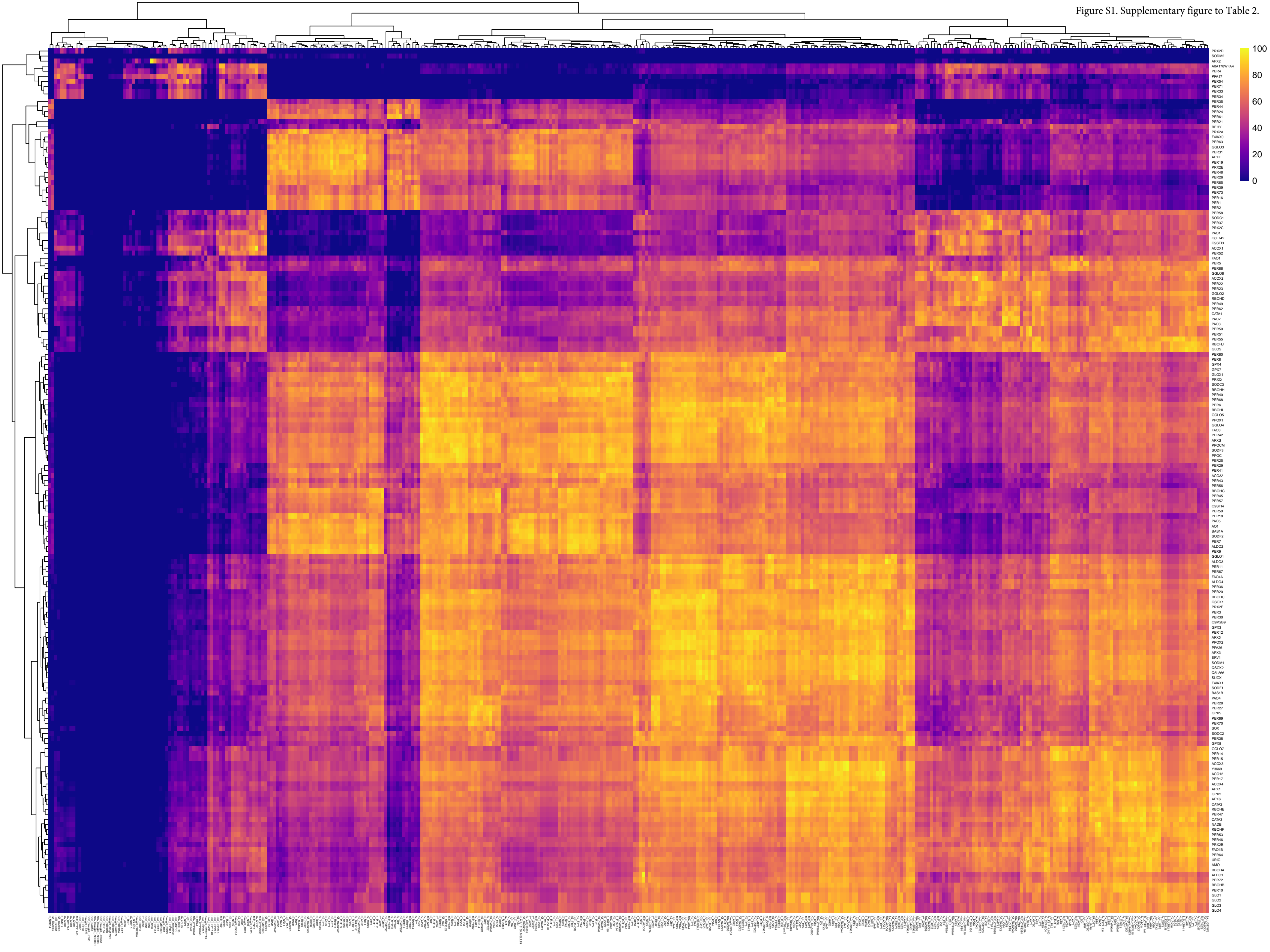

Supplement: Supplementary file 1 [file ijms-19-02812-s001.zip › Supplementary_FigureS1.pdf]
